# A Machine Learning–Based Preclinical Osteoporosis Screening Tool (POST): Model Development and Validation Study

**DOI:** 10.2196/46791

**Published:** 2023-11-08

**Authors:** Qingling Yang, Huilin Cheng, Jing Qin, Alice Yuen Loke, Fei Wan Ngai, Ka Chun Chong, Dexing Zhang, Yang Gao, Harry Haoxiang Wang, Zhaomin Liu, Chun Hao, Yao Jie Xie

**Affiliations:** 1 School of Nursing, Faculty of Health and Social Sciences, The Hong Kong Polytechnic University, Hong Kong SAR, China; 2 The Jockey Club School of Public Health and Primary Care, The Chinese University of Hong Kong, Hong Kong SAR, China; 3 Department of Sport, Physical Education and Health, Hong Kong Baptist University, Hong Kong SAR, China; 4 School of Public Health, Sun Yat-Sen University, Guangzhou, China; 5 College of Medicine and Veterinary Medicine, The University of Edinburgh, Edinburgh, United Kingdom; 6 Sun Yat‑Sen Global Health Institute, Institute of State Governance, Sun Yat-Sen University, Guangzhou, China

**Keywords:** osteoporosis, machine learning, screening tool, older people, health care, Hong Kong

## Abstract

**Background:**

Identifying persons with a high risk of developing osteoporosis and preventing the occurrence of the first fracture is a health care priority. Most existing osteoporosis screening tools have high sensitivity but relatively low specificity.

**Objective:**

We aimed to develop an easily accessible and high-performance preclinical risk screening tool for osteoporosis using a machine learning–based method among the Hong Kong Chinese population.

**Methods:**

Participants aged 45 years or older were enrolled from 6 clinics in the 3 major districts of Hong Kong. The potential risk factors for osteoporosis were collected through a validated, self-administered questionnaire and then filtered using a machine learning–based method. Bone mineral density was measured with dual-energy x-ray absorptiometry at the clinics; osteoporosis was defined as a *t* score of −2.5 or lower. We constructed machine learning models, including gradient boosting machines, support vector machines, and naive Bayes, as well as the commonly used logistic regression models, for the prediction of osteoporosis. The best-performing model was chosen as the final tool, named the Preclinical Osteoporosis Screening Tool (POST). Model performance was evaluated by the area under the receiver operating characteristic curve (AUC) and other metrics.

**Results:**

Among the 800 participants enrolled in this study, the prevalence of osteoporosis was 10.6% (n=85). The machine learning–based Boruta algorithm identified 15 significantly important predictors from the 113 potential risk factors. Seven variables were further selected based on their accessibility and convenience for daily self-assessment and health care practice, including age, gender, education level, decreased body height, BMI, number of teeth lost, and the intake of vitamin D supplements, to construct the POST. The AUC of the POST was 0.86 and the sensitivity, specificity, and accuracy were all 0.83. The positive predictive value, negative predictive value, and *F*_1_-score were 0.41, 0.98, and 0.56, respectively.

**Conclusions:**

The machine learning–based POST was conveniently accessible and exhibited accurate discriminative capabilities for the prediction of osteoporosis; it might be useful to guide population-based preclinical screening of osteoporosis and clinical decision-making.

## Introduction

Osteoporosis is a systemic skeletal disease characterized by low bone mass and microarchitectural deterioration of bone tissue, leading to enhanced bone fragility and a consequent increase in fracture risk [1]. Over 200 million people worldwide are affected by osteoporosis [2]. In Hong Kong, about 300,000 postmenopausal women and 100,000 men aged older than 50 years had osteoporosis in 2009, and the number of individuals with low bone mass is expected to double by the year 2030 [3]. The acute hospital care cost of hip fractures amounted to 1% of the total hospital budget in Hong Kong [3]. Individuals with an osteoporotic fracture may experience long-term chronic pain and disability, loss of independence, and negative psychosocial impact, resulting in decreased quality of life [4,5]. Even upon recovery, the irreversible structural loss in bone microarchitecture makes the restoration of full bone strength unlikely. Therefore, identifying persons at the highest risk of developing osteoporosis and preventing the occurrence of the first fracture is a health care priority.

The gold standard for the diagnosis of osteoporosis is the measurement of bone mineral density (BMD) with dual-energy x-ray absorptiometry (DXA) at the anteroposterior spine (lumbar 1 to 4) and left proximal femur. Unfortunately, BMD testing of an entire aging population is impossible, and DXA machines are not readily available in all lab settings due to the cost and space requirements. In Hong Kong, DXA is generally done only with a doctor’s referral for those who have a significant risk of developing osteoporosis. Moreover, the cost of a DXA scan is not generally covered by the health checkup package. These factors often lead to referrals for DXA scans being highly selective and sometimes lacking, resulting in underdiagnosis and delayed treatment of osteoporosis in affected adults. Thus, implementing early preclinical screening for targeted DXA testing for those who are at risk of osteoporosis is needed, as this would enhance timely treatment and improve the cost-effectiveness of DXA use [6]. In fact, screening for osteoporosis to reduce bone fractures is recommended by many national clinical practice guidelines [7].

Hong Kong, as a special administrative region of China, is one of the world’s most prominent financial centers, with a unique cultural background that blends Chinese and Western influences. Although several preclinical screening tools have been developed for predicting low BMD or osteoporosis [8-17], accurate preclinical prediction of osteoporosis for the Hong Kong Chinese population still represents an unmet need. Most existing preclinical screening tools are derived from ones used in Western countries [8-15], limiting the applicability to the Chinese population. Furthermore, most tools have high sensitivity but with the trade-off of relatively low specificity [8-12,16,17], which might lead to the unnecessary cost of DXA tests that return false positives. In addition, most osteoporosis predictor selection and prediction model construction has been conducted with traditional statistical techniques (eg, linear regression, logistic regression) which are constrained by the need to meet assumptions such as normal distribution and linearity. Machine learning is a method of big data analysis that automates analytical model building. It is a branch of artificial intelligence based on the idea that computers can learn from data, identify patterns, and make decisions with minimal human intervention [18]. Machine learning methods could overcome some of the limitations of traditional statistical techniques by applying computer algorithms to large, multidimensional data and can enable more precise predictor selection and improve the performance of osteoporosis risk screening tools. However, most current machine learning–based osteoporosis predictive models for the Chinese population have been built based on clinical characteristics, such as computed tomography images, radiographs, ultrasound radiofrequency signals, and molecular and genetic biomarkers [19-26], limiting their use in daily self-assessment and preclinical conditions. Therefore, in this study, we aimed to apply machine learning algorithms for osteoporosis predictor selection and prediction model construction to develop and validate an easily accessible and high-performance tool, the Preclinical Osteoporosis Screening Tool (POST), for the Hong Kong Chinese population.

## Methods

### Study Design and Setting

This study had 3 parts. First, we developed a questionnaire for data collection of potential risk factors for osteoporosis. Second, we conducted a cross-sectional study to collect data on osteoporosis as measured by BMD and potential risk factors in a sample of Chinese participants in Hong Kong. Third, we used machine learning algorithms for predictor selection and model construction and selected the best-performing model. This cross-sectional study was conducted by the Family Planning Association of Hong Kong (FPAHK) between December 2018 and December 2019. The FPAHK is a nonprofit organization in Hong Kong that provides medical and counseling services for Hong Kong residents. It offers bone health consultation, bone density measurement, laboratory tests, and treatment for men and women. Six FPAHK clinics located in Wanchai, Ma Tau Chung, Yuen Long, Tai Wai, Tsuen Wan, and Wong Tai Sin in the 3 main districts of Hong Kong provided a relatively representative sample for this study.

### Ethical Considerations

The study protocol was approved by the Ethics Committee of FPAHK and Hong Kong Polytechnic University (HSEARS20180315010). Written informed consent and permission for accessing DXA data were obtained from all participants.

### Participants

People who received the services of FPAHK were invited to participate in this study. Interested persons were screened according to the inclusion and exclusion criteria. The inclusion criteria were (1) being aged 45 years or less; (2) being a Chinese person who can speak, read, and write Chinese; (3) having had a DXA scan done at FPAHK in the past year, or planning to have one done within the next 1.5 years (DXA scans are mostly done once a year); (4) having an accessible email address for contact; (5) being willing to participate in the study and provide informed consent; and (6) providing written permission for accessing DXA data. Exclusion criteria were (1) currently being pregnant; (2) having a history or evidence of metabolic bone disease (including, but not limited to, Paget disease, osteomalacia, renal osteodystrophy or osteogenesis imperfecta); (3) having cancer(s) with known metastasis to bone.

### Questionnaire for Risk Factor Identification

A self-administered questionnaire was designed to measure osteoporosis-associated risk factors among the study population. Risk factors that were reported to have significant associations with BMD, fractures, osteoporosis, or osteopenia were collected in the preliminary version of the questionnaire [8-13,16,17,27-31]. To validate the questionnaire, 20 experts, including 6 epidemiologists, 6 doctors, 4 senior nurses, and 4 senior researchers, with rich experience in osteoporosis treatment and research were invited to rate the relevance of all the questionnaire items on a 4-point scale: 1=not relevant, 2=somewhat relevant, 3=quite relevant, and 4=highly relevant. The Content Validity Index for Items (I-CVI) was computed as the number of experts giving a rating of 3 or 4, divided by the total number of experts. The optimal value of I-CVI ranges from 0.78 to 1.00 [32]. To identify more possible risk factors for osteoporosis in our study, items with I-CVI values that were equal to or greater than 0.50 were retained in the questionnaire.

In total, 138 items were selected for the measurement of initial potential risk factors for BMD, fractures, osteoporosis, or osteopenia. Of these items, 103 had an I-CVI that was equal to or greater than 0.78. Among these 103 items, 3 were excluded because of the difficulty in obtaining answers, leading to 100 items being retained in the questionnaire. Of 35 items with an I-CVI less than 0.78, 20 were excluded directly from the questionnaire, and 15 were amended and then retained in the questionnaire according to the suggestions of the experts. Finally, 115 items were identified as potential risk factor measurements in the questionnaire.

The final questionnaire was composed of 8 sections, including (1) sociodemographic information (eg, gender, age); (2) anthropometric variables (eg, height, weight, height loss); (3) personal characteristics (eg, tooth loss); (4) reproductive health information (eg, menstruation, menopausal stage, pregnancy history, estrogen use); (5) medical and health information (eg, chronic disease, long-term medication use, surgical history, previous bone fracture); (6) lifestyle factors (eg, smoking, alcohol consumption, physical activity, sedentary behavior, sun exposure level); (7) dietary intake (eg, calcium-rich food frequency; calcium supplementation, if any; caffeine intake); and (8) family history (eg, osteoporosis among blood relatives) (Table S1 in Multimedia Appendix 1).

### Osteoporosis Identification

The BMD data were obtained from FPAHK’s medical record databases. BMD was measured with the QDR 4500 W bone densitometer (Hologic) at the lumbar spine (L2-L4) and left femoral neck. A World Health Organization study group [33] has defined osteoporosis as a *t* score (a comparison of a subject’s BMD to that of a healthy young man or woman at the particular anatomic site) more than 2.5 SD below the mean for typical young adults (*t* score ≤−2.5).

### Data Analysis Strategy

#### Predictor Selection

Predictor selection was conducted to optimize the number of predictors included in the osteoporosis prediction models. We used a 3-step strategy to select the optimal predictors. In the first step, univariate logistic regression was used to perform a preliminary screening of all variables collected from the questionnaire. Variables with *P* values less than .10 in the univariate logistic regression were selected as the initial predictors for the next step. This more liberal *P* value threshold allowed for a wider inclusion of potential predictors [34]. The random forest imputation method was used to impute the missing values in the filtered predictors in this step [35]. The percentages of missing values ranged from 0% to 13.2% (Table S2 in Multimedia Appendix 1). In the second step, a machine learning–based variable selection algorithm called Boruta was used to confirm the significantly important predictors. The algorithm is designed as a wrapper around a random forest classification algorithm that can output variable importance, which is a metric that indicates how much each variable contributes to the model prediction. Boruta iteratively compares the importance of variables with the importance of shadow variables created by shuffling the original ones. Variables that have significantly worse importance than shadows are considered unimportant variables and are consequently dropped. On the other hand, variables that are significantly better than shadows are considered important variables [36]. The important predictors confirmed by the Boruta algorithm were used to construct the full models. In the third step, we further selected the predictors based on their accessibility and convenience for daily self-assessment and health care practice. These artificially selected variables were used to construct the simplified models in the study. Considering the model complexity and practical applicability, the best-performing model was chosen from the simplified models.

#### Model Development and Validation

The data set was randomly split into a training set (70%) and a testing set (30%). The training set was used to train the prediction models and tune the parameters, while the testing set was used to test the performance of the developed models. We used several algorithms to construct the prediction models for osteoporosis, including the following machine learning models: gradient boosting machine (GBM), support vector machine (SVM), and naive Bayes (NB) (Multimedia Appendix 1)**,** as well as a commonly used algorithm, logistic regression, for comparison. The area under the receiver operating characteristic (ROC) curve (AUC) of the testing set was used to measure the model discrimination. Sensitivity, specificity, positive predictive value, negative predictive value, accuracy, and *F*_1_-score were calculated based on the optimal point of the ROC curves that had the maximal sensitivity and 1–specificity. This cutoff point was used for osteoporosis risk stratification, with those having scores above the cutoff point being classified as high risk and those below as low risk. The metric calculation formulas are provided in Multimedia Appendix 1. The best-performing model, which optimized the AUC, sensitivity, and specificity from the simplified models, was selected as the POST, whose output is a risk score for osteoporosis. Additionally, we merged the training and testing sets into a single pooled data set of 800 participants. Calibration was evaluated graphically by plotting the observed and predicted probability stratified by the deciles of the predictive risks in the pooled data set.

#### Statistical Analysis

Continuous variables are presented as mean and SD, while categorical variables are reported as numbers and percentages. A 2-tailed *t* test was used to examine difference between continuous variables and chi-squared test was used for categorical variables. All the statistical analyses were conducted using R (version 4.2.1; R Foundation for Statistical Computing). A 2-sided *P*<.05 was considered statistically significant.

## Results

The characteristics of the potential predictors collected from the questionnaire are summarized in Table S1 in Multimedia Appendix 1. The mean age of the participants was 61.30 (SD 7.65) years, 73.3% (586/800) were women, 53.5% (428/800) had obtained a tertiary or higher degree, and 39.4% (315/800) were employed (Table S1 in Multimedia Appendix 1). The overall prevalence of osteoporosis was 10.6% (85/800); the prevalence was 0.9% (2/214) for men and 14.2% (83/586) for women.

**Figure 1.**
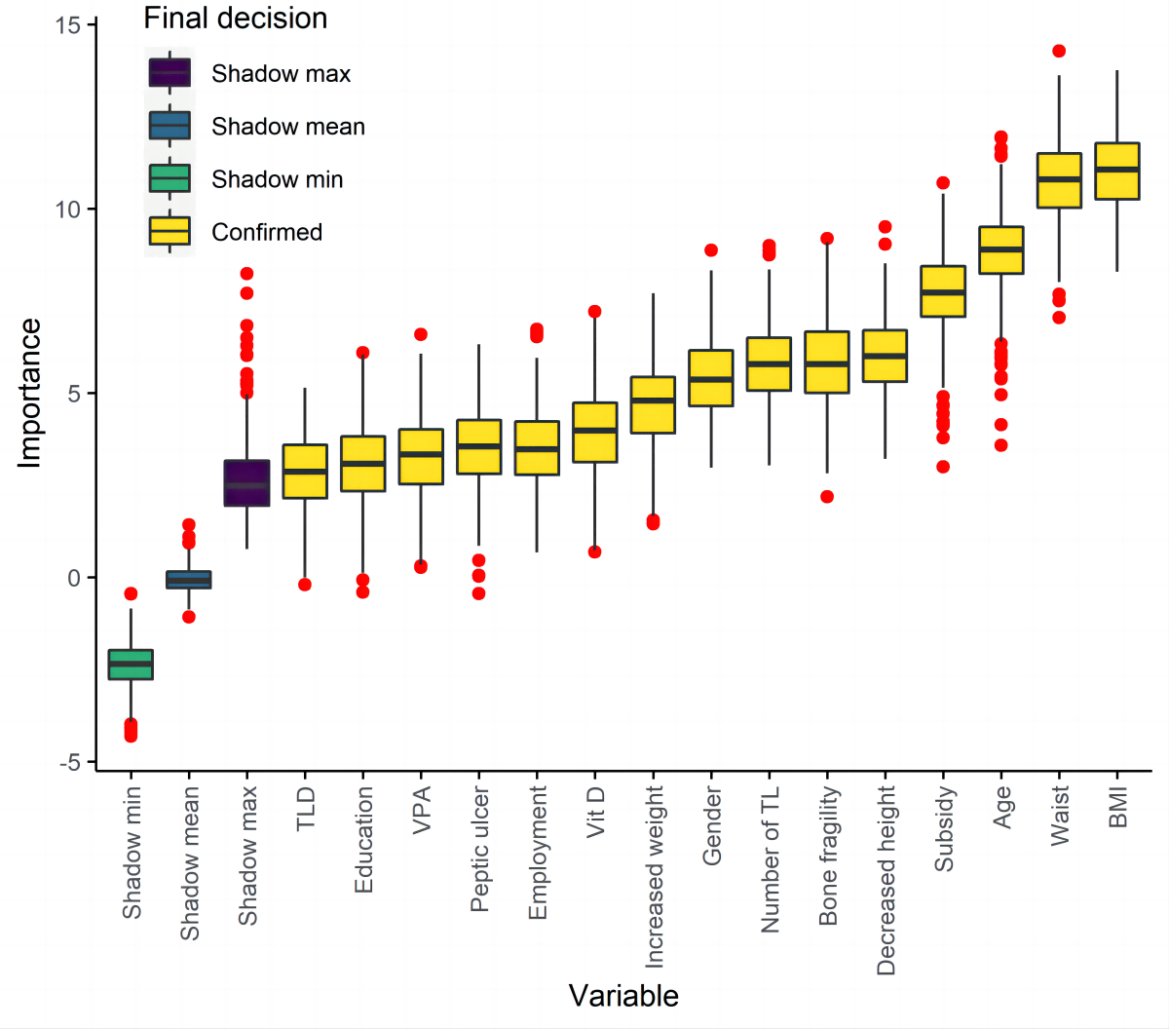
Importance of shadow variables and predictors selected by the Boruta algorithm. Shadow variables were obtained by shuffling the values of the original variables across participants and served as an external reference to decide whether the importance of any given original variable was significant; shadow max, shadow mean, and shadow min represent the maximum, mean, and minimum important values among shadow variables, respectively; variables confirmed as important were those with an importance significantly higher than the maximum shadow variable importance. TL: teeth lost; TLD: tooth loss caused by disease; Vit D: intake of vitamin D supplements; VPA: vigorous physical activity.

In the feature selection process, univariate logistic regression identified 45 potential predictors (*P*<.10), which were subsequently used as input variables for the Boruta algorithm (Table S1 in Multimedia Appendix 1). The Boruta algorithm confirmed 15 variables as significantly important predictors, with BMI, waist circumference age, social subsidy, decreased body height, bone fragility, number of teeth lost, gender, increased body weight, the intake of vitamin D supplements, peptic ulcer, employment status, vigorous physical activity, education level, and tooth loss caused by disease being ranked in order of feature importance (Figure 1). Among these variables, age, gender, education level, decreased body height, BMI, number of teeth lost, and the intake of vitamin D supplements were further selected based on their accessibility and convenience for daily self-assessment and health care practice. Compared with participants without osteoporosis, participants with osteoporosis tended to be older, have decreased more in height, have lower BMI, and have lost more teeth, were more likely to be women and to take vitamin D supplements, and were less likely to have a tertiary or higher degree (Table S1 in Multimedia Appendix 1).

**Figure 2.**
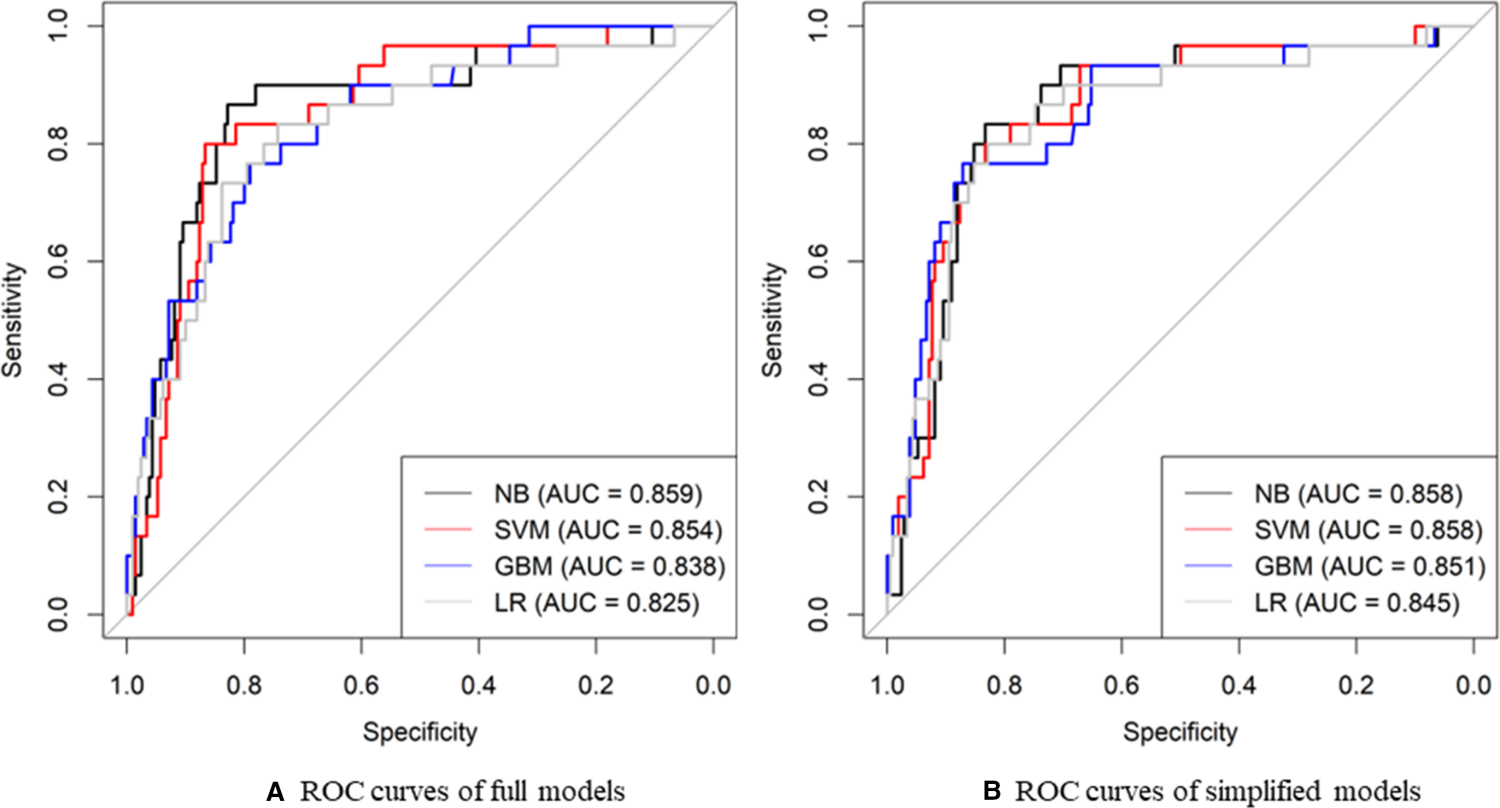
ROC curves and AUC values for osteoporosis for (A) full models and (B) simplified models. Higher AUC indicates better discrimination. Full models were constructed with the 15 predictors selected by the Boruta algorithm, including age, gender, education level, employment status, social subsidy status, decreased body height, increased body weight, BMI, waist circumference, number of teeth lost, bone fragility, peptic ulcer, vigorous physical activity, and intake of vitamin D supplements. Simplified models were constructed with the 7 predictors selected based on their accessibility and convenience for daily self-assessment and health care practice from the predictors selected by the Boruta algorithm, including age, gender, education level, decreased body height, BMI, number of teeth lost, and the intake of vitamin D supplements. AUC: area under the receiver operating characteristic curve; GBM: gradient boosting machine; LR: logistic regression; NB: naive Bayes; ROC: receiver operating characteristic; SVM: support vector machine.

The training set and testing set consisted of 560 (70%) and 240 (30%) participants, respectively. All the selected predictors and osteoporosis status were balanced between the training and testing set (Table 1). Figure 2 shows the discriminative performance of the predictive models based on ROC curves in the testing set. Among the full models, the NB model had the highest AUC at 0.859, while the LR model had the lowest AUC at 0.825 (Figure 2). A similar pattern was observed in the simplified models, where the NB model and SVM model achieved the highest AUC at 0.858 and the LR model had the lowest AUC at 0.845 (Figure 2). A comparison of the full models and simplified models with different algorithms in terms of other performance metrics is shown in Table 2. The NB model had the highest sensitivity at 0.87 for the full model and 0.83 for the simplified model (Table 2). The SVM model had the highest specificity at 0.86 for the full model, and the GBM model had the highest specificity at 0.87 for the simplified model (Table 2). Considering AUC, sensitivity, and specificity, the NB simplified model was identified as the best-performing model (hereby referred to as the POST).

**Table 1.** Characteristics of the predictors for full models and simplified models for the training and testing sets. *P* values were calculated with the chi-square test for categorical variables and a 2-tailed *t* test for continuous variables.

		Training set (n=560)	Testing set (n=240)	*P* value
Age^a^ (years), mean (SD)		61.41 (7.61)	61.06 (7.75)	.56
Women, n (%)^a^		407 (72.7)	179 (74.6)	.64
**Education level, n (%)** ^**a**^				.56
	Primary and above	69 (12.3)	31 (12.9)	
	Secondary	197 (35.2)	75 (31.2)	
	Tertiary or above	294 (52.5)	134 (55.8)	
**Employment status, n (%)**				.76
	Employed	216 (38.6)	99 (41.2)	
	Retired	223 (39.8)	90 (37.5)	
	Other (eg, housewife, unemployed)	121 (21.6)	51 (21.2)	
Receives social subsidy, n (%)		87 (15.5)	38 (15.8)	>.99
Decreased body height (cm)^a^^,b^, mean (SD)		1.47 (1.83)	1.64 (2.26)	.26
Increased body weight (kg)^c^, mean (SD)		8.17 (6.79)	8.05 (6.99)	.81
BMI (kg/m^2^)^a^, mean SD		23.15 (3.24)	22.85 (3.36)	.23
Waist circumference (cm), mean (SD)		78.36 (8.92)	77.21 (8.87)	.09
Number of teeth lost^a^, mean (SD)		2.18 (4.97)	1.85 (4.43)	.38
Tooth loss from disease, n (%)		199 (35.5)	84 (35)	.95
Bone fragility, n (%)		24 (4.3)	11 (4.6)	>.99
Peptic ulcer, n (%)		43 (7.7)	17 (7.1)	.88
Vigorous physical activity, n (%)		160 (28.6)	60 (25)	.34
Intake of vitamin D supplements, n (%)^a^		158 (28.2)	60 (25)	.40
Osteoporosis, n (%)		55 (9.8)	30 (12.5)	.32

a Predictors used only in simplified models.

b Calculated by subtracting the current height from the past highest height.

c Calculated by subtracting the past lightest weight from the current weight.

**Table 2.** Performance metrics of full models and simplified models trained by the 4 different algorithms. Full models were constructed with the 15 predictors selected by the Boruta algorithm, including age, gender, education level, employment status, social subsidy status, decreased body height, increased body weight, BMI, waist circumference, number of teeth lost, bone fragility, peptic ulcer, vigorous physical activity, and intake of vitamin D supplements. Simplified models were constructed with the 7 predictors selected based on their accessibility and convenience for daily self-assessment and health care practice from the predictors selected by the Boruta algorithm, including age, gender, education level, decreased body height, BMI, number of teeth lost, and intake of vitamin D supplements. The cutoff point was the optimal point on the receiver operating characteristic curve that could obtain maximal sensitivity and 1–specificity.

	Naive Bayes		Linear Regression		Gradient boosting machine		Support vector machine	
	Full models	Simplified models	Full models	Simplified models	Full models	Simplified models	Full models	Simplified models
Accuracy	0.83	0.83	0.75	0.83	0.79	0.85	0.85	0.83
Sensitivity	0.87	0.83	0.83	0.80	0.77	0.73	0.80	0.80
Specificity	0.82	0.83	0.74	0.83	0.79	0.87	0.86	0.83
Positive predictive value	0.41	0.42	0.32	0.40	0.34	0.45	0.45	0.41
Negative predictive value	0.98	0.97	0.97	0.97	0.96	0.96	0.97	0.97
*F*_1_-score	0.56	0.56	0.46	0.53	0.47	0.56	0.58	0.54
Cutoff point	0.20	0.11	0.09	0.15	0.11	0.18	0.09	0.10

**Figure 3.**
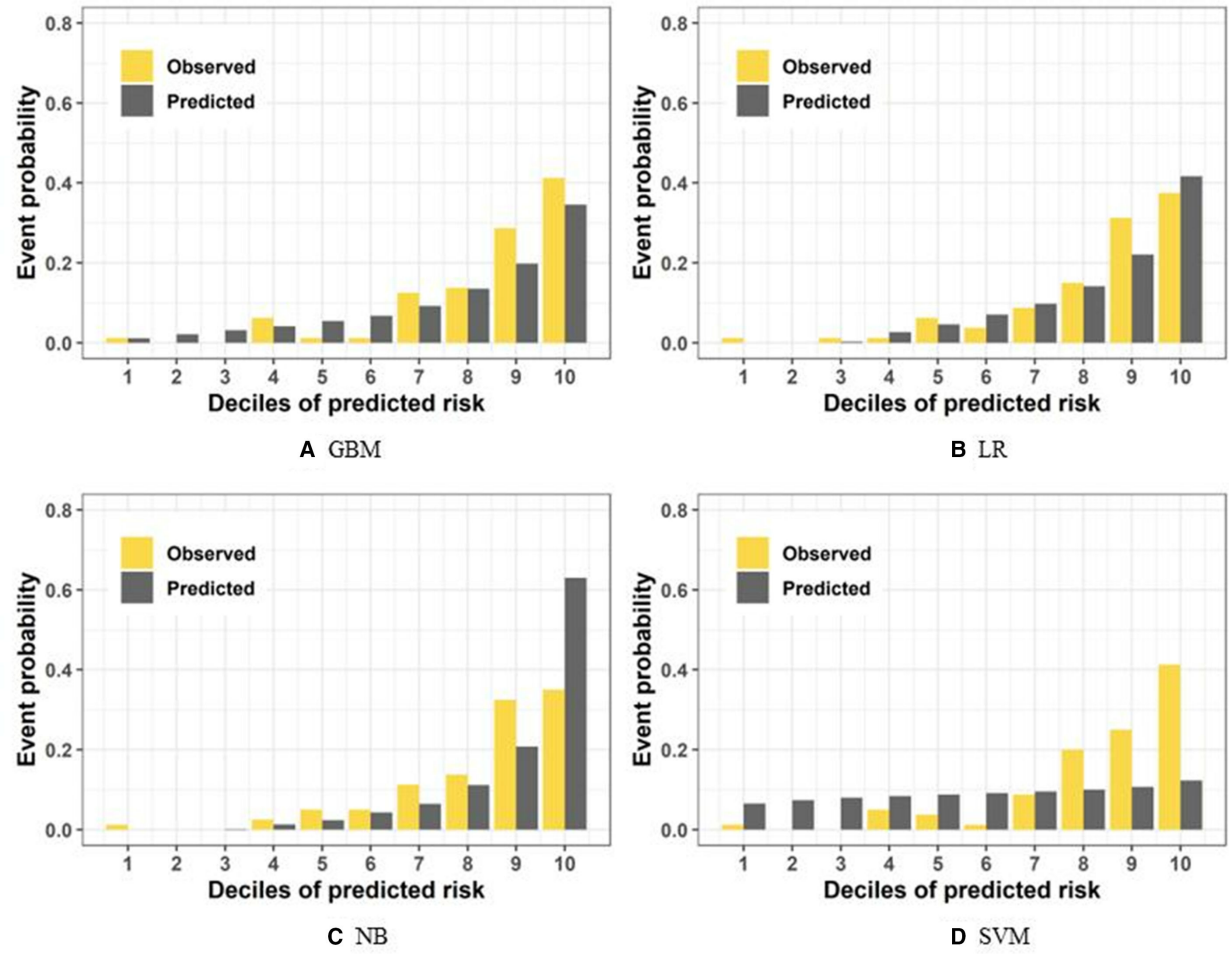
Risk of observed osteoporosis according to deciles of predicted risk of simplified models, including (A) GBM, (B), LR, (C), NB, and (D) SVM. Simplified models were constructed with the 7 predictors selected based on their accessibility and convenience for daily self-assessment and health care practice from the predictors selected by the Boruta algorithm; they included age, gender, education level, decreased body height, BMI, number of teeth lost, and the intake of vitamin D supplements. GBM: gradient boosting machine; LR: logistic regression; NB: naive Bayes; SVM: support vector machine.

The calibration bar plots show the observed vs predicted decile risks of full models and simplified models (Figure S1 in Multimedia Appendix 1 and Figure 3). For the full models, the predicted risk and the observed risk fitted well in LR, while the GBM slightly underestimated the highest risk decile and NB and the SVM overestimated the highest risk decile (Figure S1 in Multimedia Appendix 1). The calibration bar plots of the simplified models showed a similar pattern to the full models, except that the SVM underestimated the highest risk decile (Figure 3).

## Discussion

### Principal Findings

In this study, we developed and validated a self-administrated questionnaire to assess potential risk factors for osteoporosis and collected data from 6 representative clinics across 3 main districts in Hong Kong. We used machine learning–based methods to confirm significantly important variables and further selected 7 predictors based on their accessibility and convenience for daily self-assessment and health care practice. A set of machine learning models was constructed to predict osteoporosis risk, and the best-performing model, the POST, was identified. The POST demonstrated accurate discriminatory abilities in predicting osteoporosis with simple inputs that make it a convenient tool for daily self-assessment and other health care practices.

Machine learning algorithms could capture high-dimensional, nonlinear relationships among predictors and provide a novel approach to the compelling requirement for accurate personalized prediction of osteoporosis. In our study, we constructed both typical machine learning models and the traditional LR model and found that machine learning models had higher AUC values compared with the LR model. Previously established osteoporosis screening scores, including Simple Calculated Osteoporosis Risk Estimation (SCORE) [10], the Osteoporosis Risk Assessment Instrument (ORAI) [12], the Osteoporotic Self-assessment Tool (OST/OSTA) [17], the Osteoporosis Index of Risk (OSIRIS) [8], Osteoporosis Prescreening Risk Assessment (OPERA) [11], the Male Osteoporosis Screening Tool (MOST), and the Male Osteoporosis Risk Estimation Score (MORES) [9], are all derived from traditional statistical models and achieve high sensitivity (78.5%-98%) with relatively low specificity (29%-64.2%). Low specificity indicates a high false positive rate, reflecting potential resource waste for subsequent DXA use. The machined learning–based POST in our study has balanced high sensitivity (83%) and specificity (83%), and therefore has the potential to be used as a cost-effective osteoporosis preclinical screening tool to determine people at high risk who require further testing using DXA.

The selection of predictors for target outcomes is a crucial step in prediction model construction research. Traditionally, a stepwise process of regression models or empirical determination has been commonly used to select predictors. However, our study sought to improve upon this practice by combining regression and machine learning methods with an empirical, knowledge-based method to select predictors. Our findings indicate that the performance of simplified models that used only 7 predictors selected consecutively by regression, the Boruta algorithm, and empirical knowledge was comparable to that of the full models, which used 15 predictors identified by the Boruta algorithm. However, the simplified models, with fewer predictors, demonstrated better applicability to health promotion practice. Our study’s predictor selection process could provide an example for future studies.

BMI, waist circumference, and age were identified as the top 3 important variables by the Boruta algorithm. Consistently, these variables have also been commonly selected and set as the parameters in previously established osteoporosis screening scores [9-17]. We selected 7 predictors based on their accessibility and convenience for daily self-assessment and health care practice from the Boruta-identified predictors to compute simplified models, including age, gender, education level, decreased body height, BMI, number of teeth lost, and the intake of vitamin D supplements. These variables are commonly found in daily life and are highly objective, allowing individuals to provide accurate values without the need for additional clinical or lab examinations. People in the high-risk group are recommended to receive a DXA scan, and confirmed osteoporosis patients should receive appropriate treatment, while people in the low-risk group could postpone a DXA scan. The application of POST is not limited to specialized health care facilities and can be extended to community health care settings, as well as at-home use. This would enable the identification of at-risk individuals who may not have access to specialized health care services, thereby improving access to timely treatment of osteoporosis patients.

### Strengths and Limitations

Our study has several strengths, including that we collected potential risk factors from the literature and constructed a self-administered questionnaire for risk factor measurement; collected the data from 6 representative clinics spanning 3 main districts in Hong Kong; used machine learning–based algorithms for both predictor selection and model construction so as to capture multidimensional, nonlinear relationships that were not limited by the statistical assumptions of traditional statistical models; and obtained data on osteoporosis outcomes with limited misclassification due to the objective and accurate measurement of BMD by DXA.

However, several important limitations should be noted. First, the machine learning algorithms are known as “black box” models because of their complexity and uninterpretability. The models cannot compute effect size to clarify relationships between predictors and outcomes. Instead, the machine learning algorithms produce variable importance values to indicate which variables are more important to the outcome. Second, the sample size of this study was relatively not large. Future studies should include more samples as model input to further improve accuracy. Third, there was an unbalanced gender distribution, with a higher proportion of women, which could have limited the representativeness of our findings in the male population and induced potential gender-related bias. Future studies should consider more balanced gender representation, potentially supplemented by gender-specific models. Fourth, this study lacked external validation, which is our next planned research project. Fifth, the POST was derived from the Chinese population in Hong Kong and may not generalize to other populations with different ethnic, economic, and cultural backgrounds.

### Conclusions

In conclusion, we used machine learning methods to develop and internally validate the POST to predict osteoporosis among the Chinese population in Hong Kong. The POST showed accurate discrimination, with potentially important implications for the optimization of osteoporosis screening and clinical decision-making.

## Supplementary material

10.2196/46791 Multimedia Appendix 1 Supplementary tables and other material.

## Abbreviations

AUC: area under the receiver operating characteristic curve
BMD: bone mineral density
DXA: dual-energy x-ray absorptiometry
FPAHK: Family Planning Association of Hong Kong
GBM: gradient boosting machine
I-CVI: Content Validity Index for Items
LR: logistic regression
MORES: Male Osteoporosis Risk Estimation Score
MOST: Male Osteoporosis Screening Tool
NB: naive Bayes
OPERA: Osteoporosis Prescreening Risk Assessment
OSIRIS: Osteoporosis Index of Risk
OST/OSTA: Osteoporotic Self-assessment Tool
POST: Preclinical Osteoporosis Screening Tool
ROC: receiver operating characteristic
SCORE: Simple Calculated Osteoporosis Risk Estimation
SVM: support vector machine
