# Supplementary material for: A Machine Learning–Based Preclinical Osteoporosis Screening Tool (POST): Model Development and Validation Study
Source: JMIR Aging. 2023 Nov 8;6:e46791. doi: 10.2196/46791 (PMC10686208; doi:10.2196/46791)
Supplement: Multimedia Appendix 1 [file aging-v6-e46791-s001.docx]

**SUPPLEMENTARY MATERIALS**

| **Supplemental Table 1. Characteristic of the collected variables and their associations with osteoporosis** | | | | | |  |  |
| --- | --- | --- | --- | --- | --- | --- | --- |
|  | Overall | Normal | Osteoporosis | OR (95% CI) | *P*-value |  |  |
| No. of participants | 800 | 715 | 85 |  |  |  |  |
| **Social demographic characteristics** | |  |  |  |  |  |  |
| Age (years) | 61.30 (7.65) | 60.94 (7.59) | 64.35 (7.58) | 1.03 (1.06, 1.09) | < 0.001 |  |  |
| Women, N (%) | 586 (73.25) | 503 (70.35) | 83 (97.65) | 4.26 (17.49, 71.76) | < 0.001 |  |  |
| Birth in Hong Kong, N (%) | 604 (75.50) | 550 (76.92) | 54 (63.53) | 1.19 (1.91, 3.08) | 0.007 |  |  |
| Residence in Hong Kong for more than 7 years, N (%) | 793 (99.62) | 708 (99.58) | 85 (100.00) | - | - |  |  |
| Married or cohabitated, N (%) | 652 (81.50) | 585 (81.82) | 67 (78.82) | 0.48 (0.83, 1.44) | 0.5 |  |  |
| Education level, N (%) |  |  |  |  |  |  |  |
| Primary and below | 100 (12.50) | 80 (11.19) | 20 (23.53) | Ref | - |  |  |
| Secondary | 272 (34.00) | 238 (33.29) | 34 (40.00) | 0.31 (0.57, 1.05) | 0.07 |  |  |
| Tertiary or above | 428 (53.50) | 397 (55.52) | 31 (36.47) | 0.17 (0.31, 0.58) | < 0.001 |  |  |
| Employment status, N (%) | |  |  |  |  |  |  |
| Employed | 315 (39.38) | 297 (41.54) | 18 (21.18) | Ref | - |  |  |
| Retired | 313 (39.12) | 273 (38.18) | 40 (47.06) | 1.35 (2.42, 4.32) | 0.003 |  |  |
| Other (Housewife, unemployed, etc.) | 172 (21.50) | 145 (20.28) | 27 (31.76) | 1.64 (3.07, 5.76) | < 0.001 |  |  |
| Monthly family income, N (%) | |  |  |  |  |  |  |
| < HKD 9999 | 223 (27.88) | 194 (27.13) | 29 (34.12) | Ref | - |  |  |
| HKD10000-29999 | 207 (25.87) | 181 (25.31) | 26 (30.59) | 0.55 (0.96, 1.69) | 0.89 |  |  |
| HKD30000-59999 | 142 (17.75) | 134 (18.74) | 8 (9.41) | 0.18 (0.40, 0.90) | 0.03 |  |  |
| HKD>=60000 | 145 (18.12) | 134 (18.74) | 11 (12.94) | 0.27 (0.55, 1.14) | 0.11 |  |  |
| Unknown | 83 (10.38) | 72 (10.07) | 11 (12.94) | 0.49 (1.02, 2.15) | 0.95 |  |  |
| Had social subsidy, N (%) | 124 (15.82) | 101 (14.43) | 23 (27.38) | 1.32 (2.24, 3.78) | 0.003 |  |  |
| **Anthropometrics** |  |  |  |  |  |  |  |
| Decreased body height (cm)^a^ | 1.51 (2.06) | 1.42 (1.98) | 2.24 (2.54) | 1.06 (1.16, 1.28) | 0.002 |  |  |
| Decreased body weight (kg)^b^ | 2.81 (3.49) | 2.69 (3.47) | 3.78 (3.54) | 1.02 (1.08, 1.14) | 0.009 |  |  |
| Increased body weight (kg)^c^ | 8.12 (7.18) | 8.53 (7.34) | 4.80 (4.56) | 0.86 (0.90, 0.94) | < 0.001 |  |  |
| Body mass index (kg/m2) | 23.06 (3.28) | 23.35 (3.22) | 20.65 (2.73) | 0.66 (0.72, 0.79) | < 0.001 |  |  |
| Waist (cm) | 78.07 (9.25) | 78.85 (9.12) | 71.35 (7.59) | 0.86 (0.89, 0.92) | < 0.001 |  |  |
| systolic blood pressure (mmHg) | 124.28 (16.29) | 124.58 (16.29) | 121.46 (16.21) | 0.97 (0.99, 1.00) | 0.13 |  |  |
| diastolic blood pressure (mmHg) | 73.63 (10.33) | 73.81 (10.33) | 71.93 (10.31) | 0.96 (0.98, 1.01) | 0.15 |  |  |
| **Body posture characteristics** | |  |  |  |  |  |  |
| Abnormal posture, N (%) | 17 (2.12) | 11 (1.54) | 6 (7.06) | 1.75 (4.86, 13.50) | 0.002 |  |  |
| Odontogenesis, N (%) | 138 (17.29) | 121 (16.95) | 17 (20.24) | 0.71 (1.24, 2.19) | 0.45 |  |  |
| Lost teeth, N (%) | 397 (49.81) | 342 (48.03) | 55 (64.71) | 1.24 (1.98, 3.17) | 0.004 |  |  |
| Number of teeth lost | 2.08 (4.81) | 1.87 (4.38) | 3.87 (7.35) | 1.02 (1.06, 1.09) | < 0.001 |  |  |
| Natural tooth loss, N (%) | 107 (13.38) | 94 (13.15) | 13 (15.29) | 0.64 (1.19, 2.24) | 0.58 |  |  |
| Tooth loss from accidental injury, N (%) | 34 (4.25) | 27 (3.78) | 7 (8.24) | 0.96 (2.29, 5.42) | 0.06 |  |  |
| Tooth loss from diseases, N (%) | 283 (35.38) | 244 (34.13) | 39 (45.88) | 1.04 (1.64, 2.58) | 0.03 |  |  |
| **Female fertility-related characteristics^d^** | |  |  |  |  |  |  |
| Age of menarche (years) | 13.29 (1.98) | 13.24 (2.00) | 13.60 (1.84) | 0.97 (1.09, 1.22) | 0.14 |  |  |
| Number of pregnancies | 1.86 (1.38) | 1.85 (1.39) | 1.88 (1.37) | 0.86 (1.01, 1.20) | 0.87 |  |  |
| Number of births | 1.53 (1.10) | 1.51 (1.09) | 1.65 (1.18) | 0.91 (1.12, 1.38) | 0.28 |  |  |
| Number of natural births | 1.25 (1.18) | 1.25 (1.16) | 1.27 (1.31) | 0.83 (1.02, 1.24) | 0.88 |  |  |
| Number of cesarean sections | 0.28 (0.62) | 0.26 (0.59) | 0.38 (0.76) | 0.94 (1.31, 1.83) | 0.12 |  |  |
| Menopause, N (%) | 535 (92.56) | 453 (91.33) | 82 (100.00) | - | 0.98 |  |  |
| Menopause caused by operation, N (%) | 70 (13.11) | 56 (12.39) | 14 (17.07) | 0.77 (1.46, 2.76) | 0.25 |  |  |
| Natural menopause, N (%) | 463 (86.54) | 396 (87.61) | 67 (80.72) | 0.32 (0.59, 1.09) | 0.09 |  |  |
| Age of menopause (years) | 50.04 (4.95) | 50.09 (4.92) | 49.78 (5.13) | 0.94 (0.99, 1.04) | 0.60 |  |  |
| Use contraceptive, N (%) | 221 (37.78) | 193 (38.45) | 28 (33.73) | 0.50 (0.82, 1.33) | 0.41 |  |  |
| Take hormone replacement therapy, N (%) | 35 (5.97) | 30 (5.96) | 5 (6.02) | 0.38 (1.01, 2.68) | 0.98 |  |  |
| Take progesterone, N (%) | 8 (1.37) | 6 (1.19) | 2 (2.41) | 0.41 (2.05, 10.31) | 0.39 |  |  |
| **Diseases history** |  |  |  |  |  |  |  |
| Fracture from a fall, N (%) | 75 (9.38) | 62 (8.67) | 13 (15.29) | 1.00 (1.90, 3.63) | 0.05 |  |  |
| Bone fragility, N (%) | 35 (4.39) | 22 (3.09) | 13 (15.29) | 2.74 (5.66, 11.72) | < 0.001 |  |  |
| Bedridden by illness, N (%) | 65 (8.12) | 59 (8.25) | 6 (7.06) | 0.35 (0.84, 2.02) | 0.7 |  |  |
| Feeling prone to fall or afraid of falling, N (%) | 170 (21.25) | 135 (18.88) | 35 (41.18) | 1.88 (3.01, 4.82) | < 0.001 |  |  |
| Frequent falls (more than once a year), N (%) | 46 (5.75) | 39 (5.45) | 7 (8.24) | 0.67 (1.56, 3.60) | 0.3 |  |  |
| Reduced grip strength, N (%) | 187 (23.38) | 162 (22.66) | 25 (29.41) | 0.86 (1.42, 2.34) | 0.17 |  |  |
| Arthralgia/swelling/infection, N (%) | 339 (42.38) | 300 (41.96) | 39 (45.88) | 0.75 (1.17, 1.84) | 0.49 |  |  |
| Lumbar pain, N (%) | 284 (35.54) | 250 (35.01) | 34 (40.00) | 0.78 (1.24, 1.96) | 0.36 |  |  |
| Cervical pain, N (%) | 241 (30.12) | 214 (29.93) | 27 (31.76) | 0.67 (1.09, 1.77) | 0.73 |  |  |
| Weakness of limbs, N (%) | 73 (9.12) | 60 (8.39) | 13 (15.29) | 1.03 (1.97, 3.76) | 0.04 |  |  |
| The limbs often cramp, N (%) | 117 (14.66) | 98 (13.74) | 19 (22.35) | 1.04 (1.81, 3.14) | 0.04 |  |  |
| Limitation of lower extremity movement, N (%) | 114 (14.27) | 101 (14.15) | 13 (15.29) | 0.59 (1.10, 2.05) | 0.77 |  |  |
| Soft nails, N (%) | 76 (9.50) | 65 (9.09) | 11 (12.94) | 0.75 (1.49, 2.94) | 0.26 |  |  |
| Nails break frequently, N (%) | 120 (15.00) | 102 (14.27) | 18 (21.18) | 0.92 (1.61, 2.83) | 0.09 |  |  |
| Loss of appetite, N (%) | 39 (4.88) | 31 (4.34) | 8 (9.41) | 1.02 (2.29, 5.16) | 0.05 |  |  |
| Peptic ulcer, N (%) | 60 (7.50) | 47 (6.57) | 13 (15.29) | 1.33 (2.57, 4.97) | 0.005 |  |  |
| Hypertension, N (%) | 208 (26.00) | 194 (27.13) | 14 (16.47) | 0.29 (0.53, 0.96) | 0.04 |  |  |
| Myocardial infarction, N (%) | 12 (1.50) | 11 (1.54) | 1 (1.18) | 0.10 (0.76, 5.97) | 0.8 |  |  |
| Chronic obstructive pulmonary disease, N (%) | 1 (0.12) | 1 (0.14) | 0 (0.00) | - | - |  |  |
| Diabetes, N (%) | 55 (6.88) | 52 (7.28) | 3 (3.53) | 0.14 (0.47, 1.53) | 0.21 |  |  |
| Tumour, N (%) | 122 (15.27) | 99 (13.87) | 23 (27.06) | 1.37 (2.30, 3.89) | 0.002 |  |  |
| Malabsorption, N (%) | 5 (0.62) | 5 (0.70) | 0 (0.00) |  |  |  |  |
| Eating disorders, N (%) | 800 (100.00) | 715 (100.00) | 85 (100.00) | - | - |  |  |
| Gastrectomy, N (%) | 1 (0.12) | 1 (0.14) | 0 (0.00) | - | - |  |  |
| Enterectomy, N (%) | 6 (0.75) | 4 (0.56) | 2 (2.35) | 0.77 (4.28, 23.74) | 0.1 |  |  |
| Chronic renal failure, N (%) | 1 (0.12) | 1 (0.14) | 0 (0.00) | - | - |  |  |
| Rheumatoid arthritis, N (%) | 15 (1.88) | 14 (1.96) | 1 (1.18) | 0.08 (0.60, 4.59) | 0.62 |  |  |
| Gout, N (%) | 32 (4.01) | 30 (4.20) | 2 (2.35) | 0.13 (0.55, 2.34) | 0.42 |  |  |
| Sporadic radiation-induced osteopenia, N (%) | 2 (0.25) | 1 (0.14) | 1 (1.18) | 0.53 (8.50, 137.16) | 0.13 |  |  |
| Hypothyroidism, N (%) | 25 (3.13) | 19 (2.66) | 6 (7.06) | 1.08 (2.78, 7.16) | 0.03 |  |  |
| hyperthyroidism, N (%) | 39 (4.88) | 36 (5.04) | 3 (3.53) | 0.21 (0.69, 2.29) | 0.54 |  |  |
| Cushing's syndrome, N (%) | 800 (100.00) | 715 (100.00) | 85 (100.00) | - | - |  |  |
| Parkinson's disease, N (%) | 1 (0.12) | 0 (0.00) | 1 (1.18) |  |  |  |  |
| Other diseases, N (%) | 91 (11.40) | 80 (11.22) | 11 (12.94) | 0.60 (1.18, 2.31) | 0.64 |  |  |
| Long-term use of steroid medications, N (%) | 23 (2.88) | 21 (2.94) | 2 (2.35) | 0.18 (0.80, 3.46) | 0.76 |  |  |
| Use other medicine, N (%) | 179 (22.38) | 160 (22.38) | 19 (22.35) | 0.58 (1.00, 1.71) | 1 |  |  |
| **Lifestyle factors** |  |  |  |  |  |  |  |
| Drinking status, N (%) |  |  |  |  |  |  |  |
| Never | 595 (74.38) | 526 (73.57) | 69 (81.18) | Ref | - |  |  |
| Former | 13 (1.62) | 13 (1.82) | 0 (0.00) | - | 0.98 |  |  |
| Current | 192 (24.00) | 176 (24.62) | 16 (18.82) | 0.39 (0.69, 1.23) | 0.21 |  |  |
| Smoking status, N (%) |  |  |  |  |  |  |  |
| Never | 759 (94.88) | 674 (94.27) | 85 (100.00) | Ref | - |  |  |
| Former | 27 (3.38) | 27 (3.78) | 0 (0.00) | - | - |  |  |
| Current | 14 (1.75) | 14 (1.96) | 0 (0.00) | - | - |  |  |
| Passive smoking, N (%) | 70 (8.81) | 62 (8.73) | 8 (9.41) | 0.50 (1.09, 2.35) | 0.83 |  |  |
| Ability to function independently, N (%) | 791 (98.88) | 709 (99.16) | 82 (96.47) | 0.06 (0.23, 0.94) | 0.04 |  |  |
| Vigorous physical activity, N (%) | 220 (27.50) | 209 (29.23) | 11 (12.94) | 0.19 (0.36, 0.69) | 0.002 |  |  |
| Moderate physical activity, N (%) | 404 (50.50) | 368 (51.47) | 36 (42.35) | 0.44 (0.69, 1.09) | 0.11 |  |  |
| Walking, N (%) | 785 (98.12) | 701 (98.04) | 84 (98.82) | 0.22 (1.68, 12.92) | 0.62 |  |  |
| Daily sedentary time (min) | 322.02 (185.67) | 330.43 (189.33) | 248.31 (128.95) | 1.00 (1.00, 1.00) | < 0.001 |  |  |
| Restrict physical activity by health problems, N (%) | | |  |  |  |  |  |
| No | 640 (80.00) | 573 (80.14) | 67 (78.82) | Ref | - |  |  |
| Not certain | 26 (3.25) | 25 (3.50) | 1 (1.18) | 0.05 (0.34, 2.56) | 0.3 |  |  |
| Yes | 134 (16.75) | 117 (16.36) | 17 (20.00) | 0.70 (1.24, 2.19) | 0.45 |  |  |
| Daily outdoor activity time (min) | 110.38 (107.55) | 110.83 (107.63) | 106.91 (107.52) | 1.00 (1.00, 1.00) | 0.76 |  |  |
| Weekly sunshine days | 3.48 (2.92) | 3.39 (2.93) | 4.25 (2.76) | 1.02 (1.11, 1.20) | 0.01 |  |  |
| Minutes of VPA weekly (min) | 49.57 (125.26) | 52.97 (130.14) | 20.94 (66.04) | 0.99 (1.00, 1.00) | 0.03 |  |  |
| Minutes of MPA weekly (min) | 119.27 (262.43) | 120.35 (251.60) | 110.08 (342.65) | 1.00 (1.00, 1.00) | 0.73 |  |  |
| Minutes of walk weekly (min) | 531.43 (616.93) | 518.89 (611.76) | 643.79 (655.33) | 1.00 (1.00, 1.00) | 0.11 |  |  |
| **Dietary experience** |  |  |  |  |  |  |  |
| Experienced hunger in adolescence | 252 (31.58) | 227 (31.79) | 25 (29.76) | 0.55 (0.91, 1.49) | 0.7 |  |  |
| Experienced dieting in Adolescence | 104 (13.02) | 98 (13.73) | 6 (7.06) | 0.20 (0.48, 1.12) | 0.09 |  |  |
| Experienced hunger aged 20-45 years | 225 (28.12) | 205 (28.67) | 20 (23.53) | 0.45 (0.77, 1.30) | 0.32 |  |  |
| Experienced dieting aged 20-45 years | 144 (18.00) | 135 (18.88) | 9 (10.59) | 0.25 (0.51, 1.04) | 0.06 |  |  |
| Experienced hunger in the past year | 202 (25.28) | 191 (26.75) | 11 (12.94) | 0.21 (0.41, 0.78) | 0.007 |  |  |
| Experienced dieting in the past year | 129 (16.15) | 123 (17.23) | 6 (7.06) | 0.16 (0.36, 0.86) | 0.02 |  |  |
| Experienced binge eating in the past year | 115 (14.41) | 109 (15.29) | 6 (7.06) | 0.18 (0.42, 0.99) | 0.05 |  |  |
| **Food consumed at least once a week** | |  |  |  |  |  |  |
| Milk, N (%) | 281 (35.21) | 241 (33.80) | 40 (47.06) | 1.11 (1.74, 2.74) | 0.02 |  |  |
| 2 slices of cheese, N (%) | 120 (15.04) | 109 (15.29) | 11 (12.94) | 0.42 (0.82, 1.60) | 0.57 |  |  |
| 1 cup crème, N (%) | 70 (8.77) | 63 (8.84) | 7 (8.24) | 0.41 (0.93, 2.09) | 0.85 |  |  |
| 1/3 brick of tofu, N (%) | 265 (33.17) | 242 (33.89) | 23 (27.06) | 0.44 (0.72, 1.20) | 0.21 |  |  |
| 1 cup soy milk with calcium, N (%) | 99 (12.41) | 88 (12.34) | 11 (12.94) | 0.54 (1.06, 2.07) | 0.87 |  |  |
| Dark green leafy vegetables, N (%) | 746 (93.37) | 668 (93.56) | 78 (91.76) | 0.33 (0.77, 1.76) | 0.53 |  |  |
| 4 mushrooms, N (%) | 185 (23.18) | 169 (23.70) | 16 (18.82) | 0.42 (0.75, 1.32) | 0.32 |  |  |
| 4 pieces of deep sea fish (salmon, tuna, etc.), N (%) | 220 (27.60) | 204 (28.61) | 16 (19.05) | 0.33 (0.59, 1.04) | 0.07 |  |  |
| 3 edible fish with bones (sardines, nine-belly fish, etc.), N (%) | 64 (8.03) | 60 (8.42) | 4 (4.76) | 0.19 (0.54, 1.54) | 0.25 |  |  |
| 3 tablespoons sesame seeds, N (%) | 160 (20.05) | 135 (18.91) | 25 (29.76) | 1.10 (1.82, 3.01) | 0.02 |  |  |
| 2 tablespoons nuts, N (%) | 321 (40.18) | 284 (39.78) | 37 (43.53) | 0.74 (1.17, 1.84) | 0.5 |  |  |
| 1 cup of tea/coffee, N (%) | 618 (77.25) | 561 (78.46) | 57 (67.06) | 0.34 (0.56, 0.91) | 0.02 |  |  |
| 1 can of Coke, N (%) | 47 (5.88) | 45 (6.29) | 2 (2.35) | 0.09 (0.36, 1.51) | 0.16 |  |  |
| Calcium supplements, N (%) | 289 (36.22) | 242 (33.94) | 47 (55.29) | 1.53 (2.41, 3.79) | < 0.001 |  |  |
| Vitamin D supplements, N (%) | 217 (27.30) | 173 (24.37) | 44 (51.76) | 2.11 (3.33, 5.27) | < 0.001 |  |  |
| Other Supplements, N (%) | 245 (31.01) | 217 (30.74) | 28 (33.33) | 0.70 (1.13, 1.82) | 0.63 |  |  |
| **Family history** |  |  |  |  |  |  |  |
| Decreased body height, N (%) | 326 (40.75) | 297 (41.54) | 29 (34.12) | 0.45 (0.73, 1.17) | 0.19 |  |  |
| Hunchback, N (%) | 137 (17.12) | 119 (16.64) | 18 (21.18) | 0.77 (1.35, 2.35) | 0.3 |  |  |
| Bone fragility, N (%) | 194 (24.25) | 178 (24.90) | 16 (18.82) | 0.40 (0.70, 1.24) | 0.22 |  |  |
| Osteoporosis, N (%) | 221 (27.62) | 199 (27.83) | 22 (25.88) | 0.54 (0.91, 1.51) | 0.7 |  |  |
| Fracture from a fall, N (%) | 178 (22.25) | 156 (21.82) | 22 (25.88) | 0.75 (1.25, 2.10) | 0.4 |  |  |

OR, Odds ratio; VPA, vigorous physical activity; MPA, Moderate physical activity.

Data are presented as mean (sd) for continuous variables, and numbers (percentages) for categorical variables. P values were calculated with Chi-square test for categorical variables and t-test for continuous variables. OR and P values were estimated by univariate logistic regression with osteoporosis as the independent variable and a single potential predictor as the dependent variable. Missing values were omitted when conducted the analysis.

^a^Calculated by subtracting the current height from the past highest height; ^b^Calculated by subtracting the current weight from the past heaviest weight; ^c^Calculated by subtracting the past lightest weight from the current weight; ^d^ Variables only for women.

**Supplemental** **Table 2. Percentage of missing data for the univariate logistic regression-selected variables**

| Variables | Number of missing data | % of missing data |
| --- | --- | --- |
| Daily sedentary time | 106 | 13.20 |
| Waist | 98 | 12.20 |
| Increased body weight^a^ | 91 | 11.40 |
| Decreased body height^b^ | 79 | 9.88 |
| Decreased body weight^c^ | 46 | 5.75 |
| Had social subsidy | 16 | 2.00 |
| Weekly sunshine days | 5 | 0.63 |
| Vitamin D supplements | 5 | 0.63 |
| Lost teeth | 3 | 0.38 |
| Bone fragility | 3 | 0.38 |
| 4 pieces of deep-sea fish (such as salmon, tuna) | 3 | 0.38 |
| The limbs often cramp | 2 | 0.25 |
| Experienced binge eating in the past year | 2 | 0.25 |
| Milk | 2 | 0.25 |
| 3 tablespoons sesame seeds | 2 | 0.25 |
| Calcium supplements | 2 | 0.25 |
| Tumour | 1 | 0.13 |
| Hypothyroidism | 1 | 0.13 |
| Experienced dieting in Adolescence | 1 | 0.13 |
| Experienced hunger in the past year | 1 | 0.13 |
| Experienced dieting in the past year | 1 | 0.13 |
| Age | 0 | 0 |
| Gender | 0 | 0 |
| Birth in Hong Kong | 0 | 0 |
| Education level | 0 | 0 |
| Employment status | 0 | 0 |
| BMI | 0 | 0 |
| Abnormal posture | 0 | 0 |
| Number of teeth lost | 0 | 0 |
| Tooth loss from accidental injury | 0 | 0 |
| Tooth loss from diseases | 0 | 0 |
| Fracture from a fall | 0 | 0 |
| Feeling prone to fall or afraid of falling | 0 | 0 |
| Weakness of limbs | 0 | 0 |
| Nails break frequently | 0 | 0 |
| Loss of appetite | 0 | 0 |
| Peptic ulcer | 0 | 0 |
| Hypertension | 0 | 0 |
| Enterectomy | 0 | 0 |
| Ability to function independently | 0 | 0 |
| VPA | 0 | 0 |
| Minutes of VPA weekly | 0 | 0 |
| Experienced dieting aged 20-45 years | 0 | 0 |
| 1 cup of tea/coffee | 0 | 0 |

VPA, vigorous physical activity

^a^Calculated by subtracting the past lightest weight from the current weight.

^b^Calculated by subtracting the current height from the past highest height.

^c^Calculated by subtracting the current weight from the past heaviest weight.

(a) GBM

(b) LR

(c) NB

(d) SVM


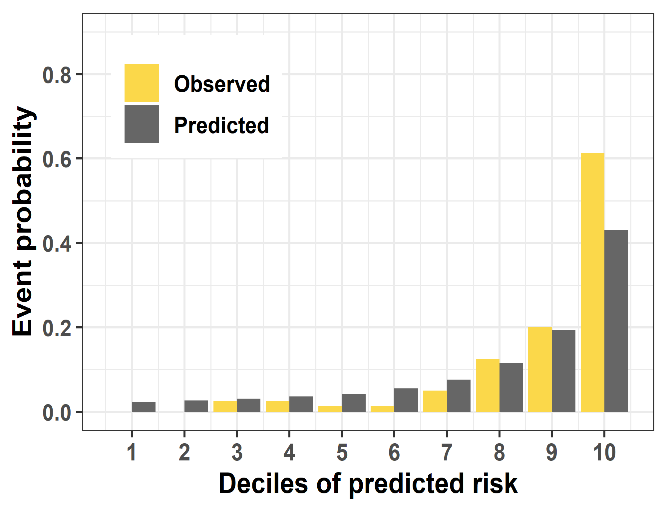

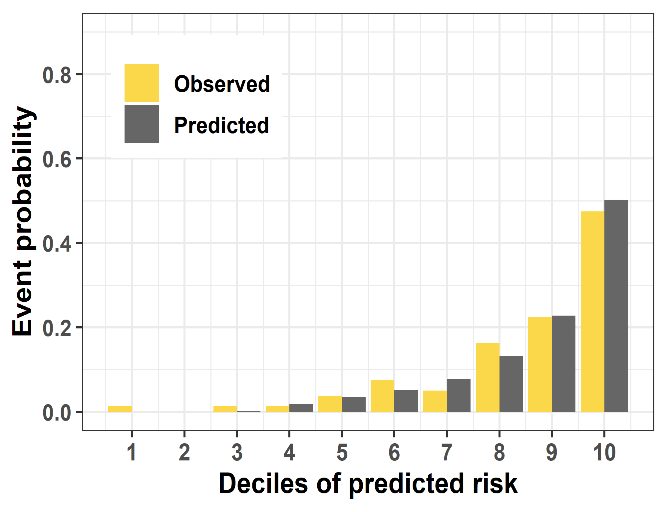

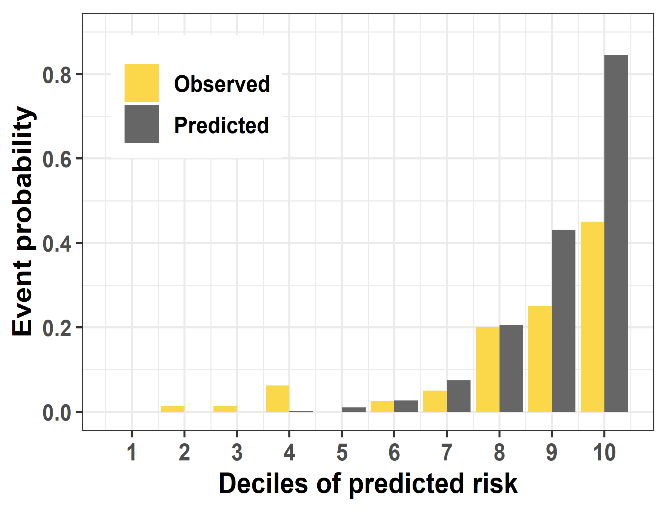

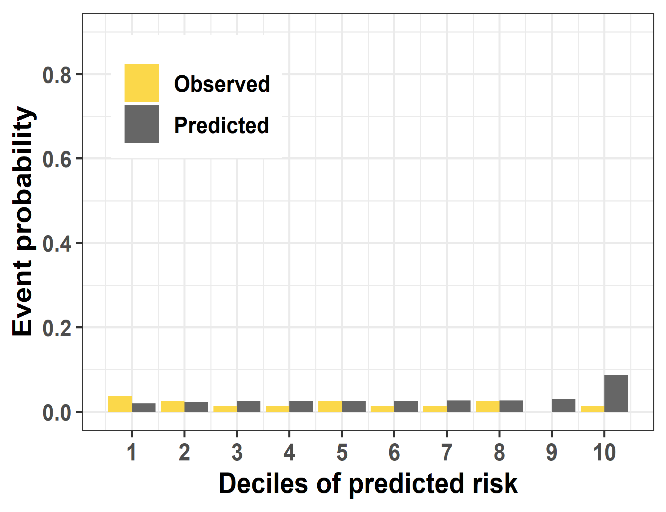


| **Supplemental Figure 1. Risk of observed osteoporosis according to deciles of predicted risk of full models.** |
| --- |
| Full models were constructed by the 15 predictors selected by the Boruta algorithm, including age, gender, education level, employment status, social subsidy status, decreased body height, increased body weight, body mass index, waist, number of teeth lost, bone fragility, peptic ulcer, vigorous physical activity, the intake of vitamin D supplements. |
| NB, naïve bayes; LR, logistic regression; GBM, gradient boosting machine; SVM, support vector machine; AUC, area under the receiver operating characteristic curve. |

**Supplemental text 1: brief description of machine learning models**

***Gradient Boosting Machine (GBM)[1]***

GBM is an ensemble machine-learning technique based on weak prediction models, which are typically decision trees. GBMs build an ensemble of shallow and weak successive decision trees with each tree learning and improving on the previous. When combined, these many weak successive trees produce a powerful “committee” that are often hard to beat with other algorithms.

***Support Vector Machine (SVM)[2]***

In the SVM algorithm, each data item is plotted as a point in n-dimensional space (where n is a number of predictors) with the value of each predictor being the value of a particular coordinate. The objective of the SVM algorithm is to find a hyperplane in n-dimensional space that distinctly classifies the data points. Support vectors are simply the coordinates of individual observation. The SVM classifier is a frontier that best segregates the two classes (hyper-plane/ line).

***Naïve Bayes (NB)[3]***

NB classifier is a probabilistic machine learning model based on Bayes’ theorem with an assumption of independence between predictors. Bayes’ Theorem provides a way that we can calculate the probability of a piece of data belonging to a given class, given our prior knowledge. It is called Naive Bayes or idiot Bayes because the calculations of the probabilities for each class are simplified to make their calculations tractable. Rather than attempting to calculate the probabilities of each attribute value, they are assumed to be conditionally independent given the class value.

**Reference**

1. Natekin, A. and A. Knoll, *Gradient boosting machines, a tutorial.* Front Neurorobot, 2013. **7**: p. 21.

2. Hearst, M.A., et al., *Support vector machines.* IEEE Intelligent Systems and their Applications, 1998. **13**(4): p. 18-28.

3. Leung, K.M.J.P.U.D.o.C.S.F. and R. Engineering, *Naive bayesian classifier.* 2007. **2007**: p. 123-156.

**Supplemental text 2: metrics calculation**

The metrics of model performance are calculated based on the four values in the cross table of observed and predicted osteoporosis status: true positive (TP), true negative (TN), False positive (FP) and false negative (FN) **(Table S4)**.

**Table S4. Cross table of observed and predicted osteoporosis status**

|  |  | Diagnosed by BMD | |
| --- | --- | --- | --- |
|  |  | Osteoporosis | Non-osteoporosis |
| Predicted by models | Osteoporosis | TP | FP |
|  | Non-osteoporosis | FN | TN |

BMD, bone mineral density;

TP, true positive; TN, true negative; FP, false positive; FN, false negative.

The formulas of the metrics used in this study are as follows.

$Accuracy=$ (TP+TN)/(TP+TN+FP+FN)

$$Sensitivity=TP/(TP+FN)$$

$$Specificity=TN/(FP+TN)$$

$Pos Pred Value=$TP/(TP+FP)

$Neg Pred Value=$TN/(FN+TN)

$$F1 score=\frac{2TP}{2TP+FN+FP}$$
